# Experimental Study on the Minimum Undeformed Chip Thickness Based on Effective Rake Angle in Micro Milling

**DOI:** 10.3390/mi11100924

**Published:** 2020-10-05

**Authors:** Xian Wu, Li Liu, Mingyang Du, Jianyun Shen, Feng Jiang, Yuan Li, Yiyang Lin

**Affiliations:** 1 College of Mechanical Engineering and Automation, Huaqiao University, Xiamen 361021, China; m15238120553@163.com (M.D.); jianyun@hqu.edu.cn (J.S.); liyuan@hqu.edu.cn (Y.L.); 2 Xiamen Tongan Vocational Technology School, Xiamen 361100, China; liuli20200930@163.com (L.L.); linyiyang88@163.com (Y.L.); 3 Institute of Manufacturing Engineering, Huaqiao University, Xiamen 361021, China; jiangfeng@hqu.edu.cn

**Keywords:** effective rake angle, micro milling, minimum undeformed chip thickness, size effect

## Abstract

Micro milling is widely used to manufacture micro parts due to its obvious advantages. The minimum undeformed chip thickness, the effective rake angle, and size effect are the typical characteristics and closely related to each other in micro milling. In this paper, the averaging method is proposed to quantitatively estimate the effective rake angle in the cutting process. The minimum undeformed chip thickness is explained based on the effective rake angle and determined to be 0.17 *r_n_* (tool cutting edge radius). Then, micro milling experiment was conducted to study the effect of the minimum undeformed chip thickness. It is found that the minimum undeformed chip thickness results in the unstable cutting process, the uneven peaks on cutting force signal, and the dense characteristic frequency distribution on frequency domain signal. The dominant ploughing effect induces the great specific cutting energy and the deteriorated surface roughness due to the minimum undeformed chip thickness.

## 1. Introduction

In recent years, the requirements for miniaturization of part and device have become increasing with the rapid development of many fields, such as communications, electronics, biomedicine, etc. With the fabrication of micro cutting tool, micro milling has been widely used to manufacture micro parts due to the obvious advantages, such as high efficiency and quality [1,2,3]. However, compared with macro milling, micro milling process presents some unique characteristics which greatly affect the machining quality. In micro milling, the machining parameters are downscaled to several microns level, and the undeformed chip thickness is comparable to the cutting edge radius of micro end mills. The tool cutting edge radius cannot be ignored and becomes an important factor [4]. The induced problems, such as the size effect, the minimum undeformed chip thickness, and effective rake angle, have significant effect on the cutting process and machining quality [5,6].

Understanding the related mechanisms in micro milling is critical for optimizing the machining process. The size effect in metal cutting which attracts much attention by researchers usually is understood as the nonlinear increase in specific cutting energy with the decrease of undeformed chip thickness [7]. Vollertsen et al. [8] classified the sources of size effects into three categories that include density effects, shape effects and microstructure effects. For micro milling, the size effect mainly is caused by the tool cutting edge radius, the change of material properties, and micro friction at the downscaling undeformed chip thickness. Aramcharoen et al. [9] studied the size effect influenced by tool geometries and found that the size effect becomes obvious once the ratio of undeformed chip thickness to tool cutting edge radius is less than one. Wu et al. [10] proposed a method to separate the ploughing force and reported that the nonlinear increase of specific cutting energy is attributed to the ploughing force. Liu and Melkote [11] found that the strain gradient strengthening at small undeformed chip thickness contributes significantly to size effect. Feng and Sagapuram [12] found the adhesion and friction at small scale tool-chip contacts are responsible for the nonlinear increase of specific cutting energy in micro cutting with approximately ideal sharp tools.

The determination of the minimum undeformed chip thickness is very important to achieve the material removal and avoid the ploughing effect in micro milling [13]. The minimum undeformed chip thickness is greatly affected by the tool geometries and material properties. There are many methods to determine the minimum undeformed chip thickness, such as the observation of the chip formation, cutting force signal, and acoustic emission signal. Shi et al. [14] reported that the minimum undeformed chip thickness is 1.4 μm (tool cutting edge radius of 6 μm) for micro milling of Inconel 718 based on the acoustic emission signal. Chen et al. [15] reported the minimum undeformed chip thickness to be 0.43–0.48 *r_n_* (tool cutting edge radius) based on the periodicity of cutting force in micro milling of potassium dihydrogen phosphate (KDP) crystal. To optimize the machining parameters, Rezaei et al. [16] found that the minimum undeformed chip thickness varies between 0.15 *r_n_* and 0.49 *r_n_* in micro milling of titanium alloy. Dib et al. [17] observed the signal variation of cutting force and found the minimum undeformed chip thickness is about 0.3 *r_n_* in micro milling of aluminum alloy 6061. Niu et al. [18] found that the minimum chip thickness for micro milling on aluminum 6082-T2 is around 0.2–0.3 μm, which is about 14–21% of tool cutting edge radius.

Another phenomenon in micro milling is that once the undeformed chip thickness is less than tool cutting edge radius, the nominal rake angle is invalid and the effective rake angle begins to work in cutting process [19]. Manjunathaiah and Endres [20] pointed out that the increase in specific cutting energy is attributed to the apparent negative effective rake angle in cutting process. Jing et al. [21] used the effective rake angle to determine the normal and frictional force components to develop cutting force model during micro milling. Yuan et al. [22] defined the effective rake angle and applied it to calculate the force coefficients, as well. In fact, the size effect, the minimum undeformed chip thickness and effective rake angle all are the typical features and closely related to each other in micro milling process. There are a lot of literatures on each of them individually, but little researches on their relationship.

In this paper, micro milling experiments are performed with the coated micro end mill. A novel calculation model is used to estimate the effective rake angle in micro milling, and the minimum undeformed chip thickness is predicted based on the effective rake angle. Then, the effect of the minimum undeformed chip thickness on the cutting process, cutting force signal, and surface roughness is studied in detail.

## 2. The Determination of Minimum Undeformed Chip Thickness Based on Effective Rake Angle

### 2.1. The Calculation Model of Effective Rake Angle

**Figure 1:**
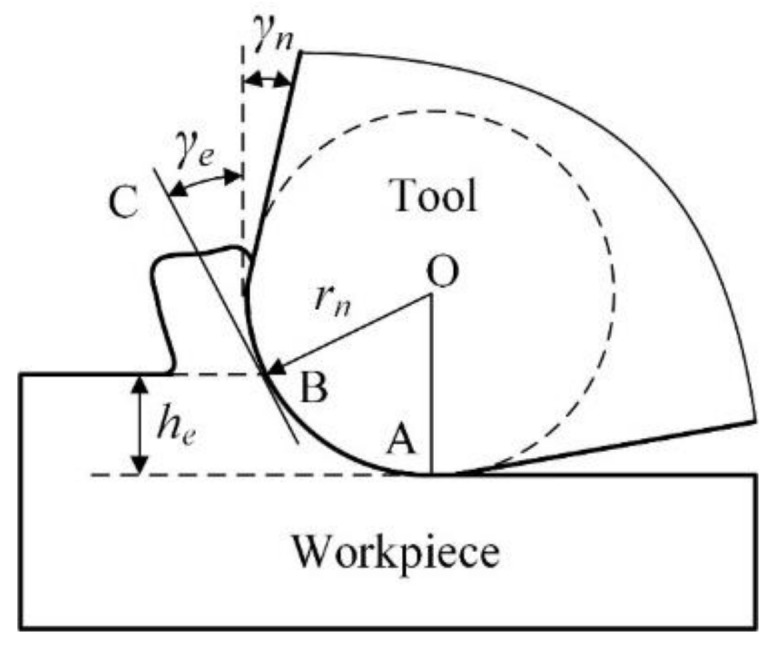
Schematic of effective rake angle.

In micro milling, the undeformed chip thickness usually is downscaled to comparable or even less than tool cutting edge radius. When the undeformed chip thickness *h_e_* is less than tool cutting edge radius *r_n_*, only the bottom arc segment of cutting edge participates in cutting process. The nominal rake angle *γ_n_* is invalid, and the cutting process gets into the state of negative rake angle. The cutting state with negative rake angle may change the stress distribution, induce the serious ploughing, and then affect machining quality. To quantitatively estimate the cutting state of negative rake angle, the effective rake angle *γ_e_* is defined. In most literatures [23], the tangent line BC at the intersection point B between workpiece surface and tool cutting edge arc is assumed to the effective rake face, as shown in Figure 1. Hence, the effective rake angle *γ_e_* is defined as the included angle between the tangent line BC and the vertical line, and it can be calculated as the following:

(1) $$\gamma_{e}=\arcsin\left(\frac{h_{e}}{r_{n}}-1\right)\;\;\;\;\;\;\;\;\;\;\;\;h_{e}\leq r_{n}$$

From this equation, it is seen that the effective rake angle mainly depends on the ratio of the undeformed chip thickness to tool cutting edge radius *h_e_*/*r_n_*. The effective rake angle at different cutting conditions is easily calculated with this equation based on the ratio of *h_e_*/*r_n_*. This calculation equation is widely used in the literatures and can be called the tangent method.

The tangent method is established on the assumption that the effective rake face is the tangent line BC. However, the actual rake face is the tool cutting edge arc AB. It is seen that the actual rake face is a curved surface, but not a plane surface. Along the tool cutting edge arc from the intersection point B to the bottom point A, the corresponding effective rake angle becomes smaller and smaller. The effective rake angle obtains the minimum value of −90° at the point A. This means that the tangent method just simply selects the maximum value of effective rake angle at the point B to quantitatively estimate the cutting state of negative rake angle. Hence, the actual negative rake angle in cutting process is greatly underestimated in the existing tangent method. For instance, at the case of *h_e_* = *r_n_*, the effective rake angle calculated by the tangent method using Equation (1) is 0°. However, the effective rake angle should be still a negative value at this case. Because the actual rake face still is the tool cutting edge arc under the nominal rake face, and so the cutting process still is in the state of negative rake angle. It is found that the effective rake angle calculated by the existing tangent method is not consistent with the actual cutting state, and this method cannot accurately estimate the cutting state of negative rake angle in micro milling.

In this paper, the novel calculation model which called the averaging method is proposed to quantitatively estimate the actual cutting state of negative rake angle in micro milling. The average value of the included angle between the tangent line and vertical line at all the point on tool cutting edge arc AB is defined as the effective rake angle. In other words, the averaging method is the average value of the tangent method at all the point on tool cutting edge arc AB. After averaging, the obtained effective rake angle is more consistent with the actual cutting state. In the averaging method, the calculation process of the effective rake angle can be divided into two steps. Firstly, the tangent method is integral on tool cutting edge arc AB from the value zero to the ratio of *h_e_*/*r_n_*, and then the integration value is divided by the ratio of *h_e_*/*r_n_* to obtain the average value. The integration on tool cutting edge arc AB is expressed as the following Equation (2), where the parameters *x* = *h_e_*/*r_n_* and C is the constant. When *x* = 0, the constant C is calculated to be −π/2. At the averaging step, the effective rake angle *γ_e_* can be calculated as the following Equation (3).

(2) $$\int \arcsin\left(x-1\right)\;dx=x\;\arcsin\left(x-1\right)-\int x\;d\left[\arcsin\left(x-1\right)\right]\;=x\;\arcsin\left(x-1\right)+\int \frac{x}{\sqrt{1-{\left(x-1\right)}^{2}}}\;d\left(x-1\right)\;\;\;\;=\left(x-1\right)\arcsin\left(x-1\right)+\sqrt{1-{\left(x-1\right)}^{2}}+C\;\;,$$

(3) $$\gamma_{e}=\frac{\int_{0}^{x}\arcsin\left(x-1\right)dx}{x}=\frac{\left(x-1\right)\arcsin\left(x-1\right)+\sqrt{1-{\left(x-1\right)}^{2}}-\frac{\pi }{2}}{x}$$

Therefore, the calculation mode of effective rake angle is expressed as the following:

(4) $$\left\{\begin{array}{c} \;\gamma_{e}=\gamma_{n}\;\;\;\;h_{e}>r_{n}\\ \;\gamma_{e}=\frac{\left(h_{e}/r_{n}-1\right)\arcsin\left(h_{e}/r_{n}-1\right)+\sqrt{1-{\left(h_{e}/r_{n}-1\right)}^{2}}-\frac{\pi }{2}}{h_{e}/r_{n}}\;\;\;\;\;\;h_{e}\leq r_{n} \end{array}\right.$$

### 2.2. The Determination of Minimum Undeformed Chip Thickness

**Figure 2:**
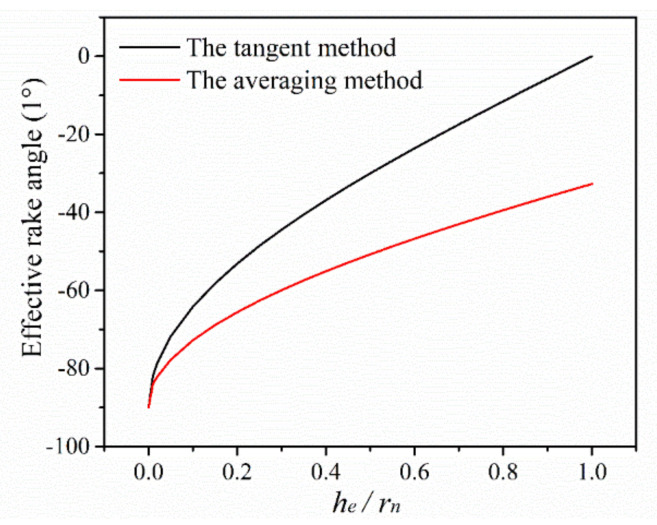
The effective rake angle varies with the ratio of *h_e_*/*r_n_*.

The comparison of effective rake angle curve obtained by the tangent method and averaging method is shown in Figure 2. It is found that the effective rake angle gradually becomes more negative with decreasing the ratio of *h_e_*/*r_n_*. It indicates that the cutting state of negative rake angle is more and more severe, the ploughing effect in cutting process becomes more serious, as well. By comparison, it is found that when the ratio of *h_e_*/*r_n_* is close to zero, the effective rake angle calculated by two methods are very approximate. But once the ratio of *h_e_*/*r_n_* is above zero, the effective rake angle calculated by the averaging method is smaller than the tangent method. The averaging method is more accurate, for example, when the ratio of *h_e_*/*r_n_* = 1, the effective rake angle calculated by the averaging method is −32.7°, which shows that the cutting process has already gone into the cutting state of negative rake angle. The result is more in accordance with the actual cutting process in micro milling.

The prediction of minimum undeformed chip thickness is a key factor for the selection of micro milling parameters. In fact, the minimum undeformed chip thickness not only is depended on the tool cutting edge geometries and material features of workpiece but also closely related with the cutting state of negative rake angle. In the cutting process with negative rake angle tool, the material flow is divided into two parts due to the stagnation point on tool cutting edge. The material above the stagnation point flows to the rake face and forms chips. The material under the stagnation point flows over tool cutting edge and remains on the machined surface. The thickness of stagnation region is increasing with more negative of tool rake angle. Since the negative rake angle reaches a critical value, the stagnation layer thickness exceeds the undeformed chip thickness, and all the material will flow to the machined surface. There is no chip formed in cutting process, and then the minimum undeformed chip thickness phenomenon occurs. This critical condition that should be satisfied for chip formation is called the minimum negative rake angle.

Komanduri [24] early found that there is no chip formation when the tool rake angle becomes more negative than −75° in orthogonal cutting of mild steel with negative rake angle tools. It indicates that the minimum negative rake angle for chip formation is −75°. Then, he also found the minimum negative rake angle is −65° in nanometric cutting of copper material [25]. Ding et al. [26] found that the minimum negative rake angle is −77° in orthogonal cutting of AL 1060. Lai et al. [27] reported that the minimum negative rake angle for chip formation of copper is between −65° and −70°. Based on the effective rake angle curve, it is obtained that when the effective rake angle is −65° and −70°, the corresponding ratio of *h_e_*/*r_n_* is 0.21 and 0.13, respectively. The averaging value of the two ratios is calculated to *h_e_*/*r_n_* = 0.17. This is the critical ratio for chip formation during micro milling of copper. Based on the existing literature, the minimum undeformed chip thickness of copper material is about *h_e_*/*r_n_* = 0.1–0.3. It indicates that the calculation result is consistent with the existing literatures. The cutting edge radius of micro end mill used in this work is 4.4 μm, and then the minimum undeformed chip thickness is predicted to *h_min_* = 0.75 μm. The minimum undeformed chip thickness has great effect on the cutting process and machining quality due to the downscaling machining parameters in micro milling.

## 3. Experimental Procedure

**Figure 3:**
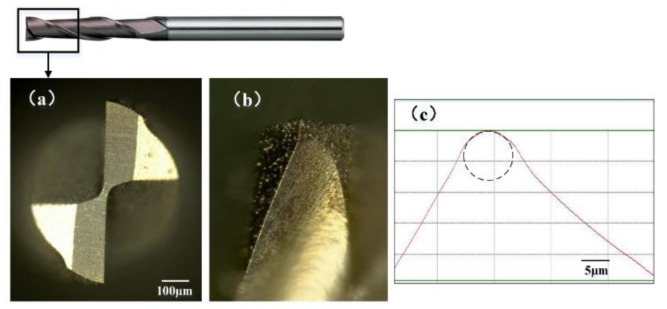
Micro end mill: (**a**) bottom view, (**b**) enlarged view, (**c**) cutting edge profile.

The tool used in this work was a coated micro end mill (MSE 230, NS tool, Tokyo, Japan) with two flutes and tool diameter of 1mm, as shown in Figure 3. The tool material and coating material are ultrafine grain cemented carbide and TiCN, respectively. The micro end mill made of cemented carbide is suitable for manufacturing micro parts that made of copper and aluminum materials [28]. The tool cutting edge length is 2.5 mm, and the helix angle is 30°. The corner radius of micro end mill is about 5.3 μm. The used micro end mill was inspected with optical microscope (VHX1000, Keyence, Osaka, Japan) to ensure that there are no micro defects on each cutting edge. After 3D profile reconstruction, the cross-sectional profile of tool cutting edge is observed, as shown in Figure 3c. With five times measuring and averaging, the tool cutting edge radius *r_n_* was measured to be about 4.4 μm.

**Figure 4:**
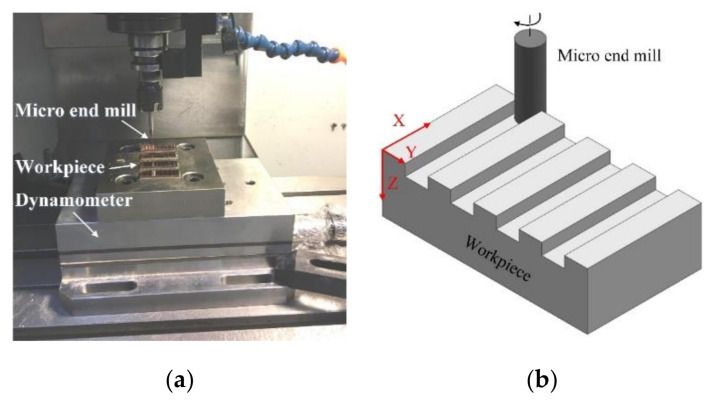
Micro milling experiment: (**a**) vertical machining center, (**b**) schematic of groove milling.

Micro milling experiments were conducted on a micro vertical machining center (OM-2A, HAAS, Oxnard, CA, USA), as shown in Figure 4. The machine tool is specially designed to manufacture micro parts. It can achieve the maximum spindle speed of 30,000 rpm and the repeated positioning accuracy of ±2 μm. The workpiece material used in this paper was pure copper T2. The workpiece samples were cut into small pieces with dimensions of 30 × 10 × 3 mm and then mounted on machine worktable. In the micro milling experiment, the cutting force was recorded using dynamometer Kistler 9257B with the sampling frequency of 20 kHz.

The schematic of straight groove milling experiments is shown in Figure 4b. First, the start surface of workpiece was machined using cemented carbide end mill with diameter of 5 mm to ensure flatness and smooth, and then the straight grooves were machined. The obtained roughness of start surface is about 0.5 μm. The detailed micro milling parameters are shown in Table 1. Nine levels of the feed per tooth contain larger and smaller than tool cutting edge radius. Each experiment was repeated three times, and the averaging values were adapted as the experimental results. After experiment, the workpiece was cleaned using an ultrasonic cleaner, and then the surface roughness Ra was measured with white light interference optical profiler (NV7300, Zygo, Middlefield, CT, USA) five times at the different positions. The measuring length is 1.4 mm, and the filter is off in surface roughness measurement.

## 4. Results and Discussion

### 4.1. The Effect of the Minimum Undeformed Chip Thickness on Cutting Process

**Figure 5:**
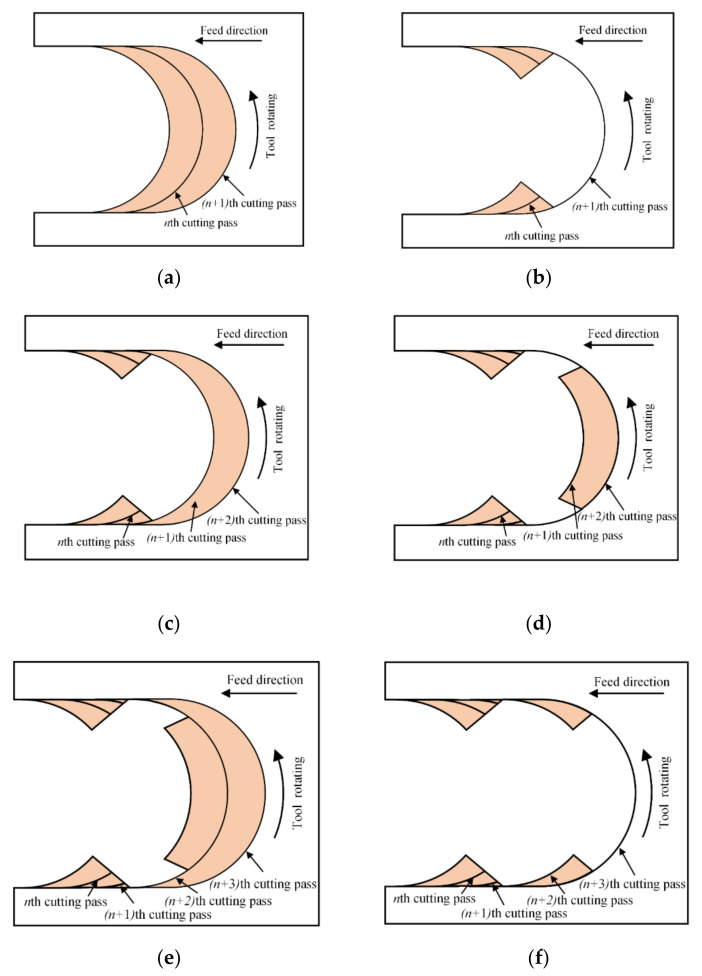
The effect of the minimum undeformed chip thickness on cutting process: (**a**) the *n*th cutting pass, (**b**) the (*n* + 1)th cutting pass, (**c**) the residual material in the (*n* + 2)th cutting pass, (**d**) the material removal in the (*n* + 2)th cutting pass, (**e**) the residual material in the (*n* + 3)th cutting pass, (**f**) the material removal in the (*n* + 3)th cutting pass.

Micro milling is a discontinuous cutting process. In each rotating period of micro end mill, there are cut-in and cut-out stage. The removed material area in single tool rotating period is a crescent shape, as shown in Figure 5a. The instantaneous undeformed chip thickness always varies with tool rotating. It gradually increases from zero to the feed per tooth in the cut-in stage, and the maximum undeformed chip thickness is obtained at the tool rotating angle of 90° and then decreases to zero in the cut-out stage. This reason induces the minimum undeformed chip thickness phenomenon occurring in both the cut-in and cut-out stage.

Since the undeformed chip thickness is very small in micro milling, the cutting process is greatly affected by the minimum undeformed chip thickness. If the feed per tooth is less than *h_min_* in the *n*th cutting pass, the tool just scratches on workpiece surface, and there is no chip formed, as shown in Figure 5a. In the (*n* + 1)th cutting pass, with the accumulation of residual material in the previous cutting pass, the actual undeformed chip thickness increases and exceeds the minimum undeformed chip thickness. But, due to the instantaneous undeformed chip thickness varying with the tool rotating, the material removal does not occur in the whole tool rotating period. At the cut-in and cut-out stage, the instantaneous undeformed chip thickness still is less than the minimum undeformed chip thickness, so there is no chip formed in the side position. Only after the tool rotates to a critical angle and the instantaneous undeformed chip thickness exceeds the minimum undeformed chip thickness can the cutting behavior occur and the chips form in the middle stage, as shown in Figure 5b. In this cutting process, there is some residual material remained on the side position of milled groove due to the deficient cutting behavior.

In the (*n* + 2)th cutting pass, the accumulated residual material on the side position of milled groove increases the instantaneous undeformed chip thickness and leads to its early achievement of the minimum undeformed chip thickness in the cut-in and cut-out stage, as shown in Figure 5c. Then, the cutting behavior and material removal occur in the cut-in and cut-out stage. But, in the middle stage of tool rotating period, the instantaneous undeformed chip thickness may still less than *h_min_* because there is no residual material. And then the cutting behavior cannot occur, and the residual material remains for the next cutting pass, as shown in Figure 5d. In the (*n* + 3)th cutting pass, the residual material only is remained on the middle position, as shown in Figure 5e. Hence, the cutting behavior cannot occur in the cut-in and cut-out stage but occur in the middle stage, and the cutting process begins to go into the next cycle, as shown in Figure 5f. This cycle of cutting process can go on occurring during the whole micro milling process. The unstable cutting behavior that caused by the minimum undeformed chip thickness phenomenon can result in the corresponding fluctuate in the cutting force signal and surface quality [29].

### 4.2. The Effect of the Minimum Undeformed Chip Thickness on Cutting Force Signal

**Figure 6:**
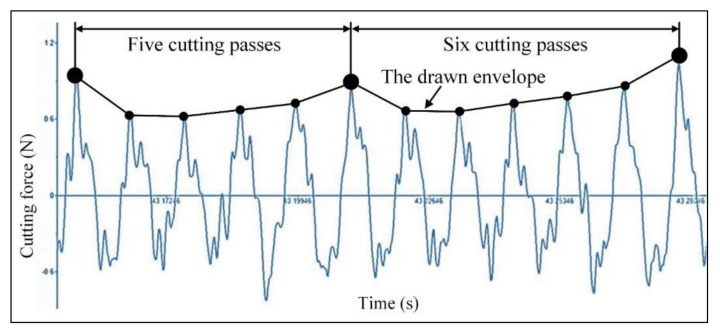
The cutting force signal waveform at *f_z_* = 0.09 μm (*a_p_* = 5 μm, *n* = 16,000 rpm).

The cutting force is an important factor for monitoring the cutting process. The cutting vibration, tool wear, and machining quality can be reflected from the cutting force signal. The cutting force signal waveform with low pass filter using the edge frequency of 600 Hz when the feed per tooth is less than *h_min_* at *f_z_* = 0.09 μm, as shown in Figure 6. The peaks observed on the cutting force signal waveform are obtained at the tool rotating angle of 90°. Each peak represents a cutting pass during micro milling process. Although these peak points exhibit very good periodicity, their amplitude values are not constant. To observe their evolution trend, the envelope of peak points on the cutting force signal waveform is connected. It is observed that the drawn envelope is not smooth, with some hump points, as well. This is the typical feature that caused by the effect of the minimum undeformed chip thickness on cutting force signal, and it also verifies the minimum undeformed chip thickness phenomenon during micro milling. When the feed per tooth is less than *h_min_*, there is no cutting behavior and material removal in the first several cutting passes. Once the actual undeformed chip thickness reaches the minimum undeformed chip thickness with the gradual accumulation of residual material in previous cutting passes, the cutting behavior and material removal can occur, and a hump point appears on the drawn envelope of the cutting force signal.

From the results in Section 2.2, the minimum undeformed chip thickness of copper material is 0.75 μm based on the effective rake angle curve. When the feed per tooth is *f_z_* = 0.09 μm, in theory, it needs about eight cutting passes to reach the minimum undeformed chip thickness. But the actual interval of the hump points on the drawn envelope is about five or six cutting passes. The reason is that the workpiece material may have elastic recovery due to the severe ploughing during cutting process. It can result in the increase of the residual material and actual undeformed chip thickness. Therefore, the actual number of cutting passes that need to be accumulated to achieve the minimum undeformed chip thickness is less than the theoretical number.

**Figure 7:**
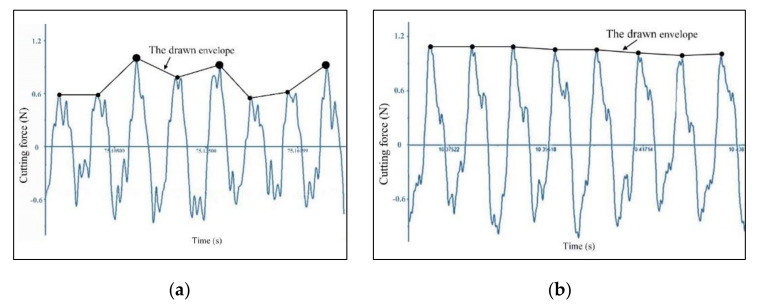
The comparison of cutting force signal waveform at different feed per tooth (*a_p_* = 5 μm, *n* = 16,000 rpm): (**a**) *f_z_* = 0.22 μm, (**b**) *f_z_* = 2.2 μm.

The comparison of cutting force signal waveform between different feeds per tooth is shown in Figure 7. It is clear to observe the effect of the minimum undeformed chip thickness on the drawn envelope. When the feed per tooth is less than *h_min_* at *f_z_* = 0.22 μm, the drawn envelope is very uneven and distributes some hump points with the interval of two or three cutting passes. Once the feed per tooth increases to larger than *h_min_* at *f_z_* = 2.2 μm, the peaks on the cutting force signal are very uniform, and the drawn envelope becomes relatively smooth.

**Figure 8:**
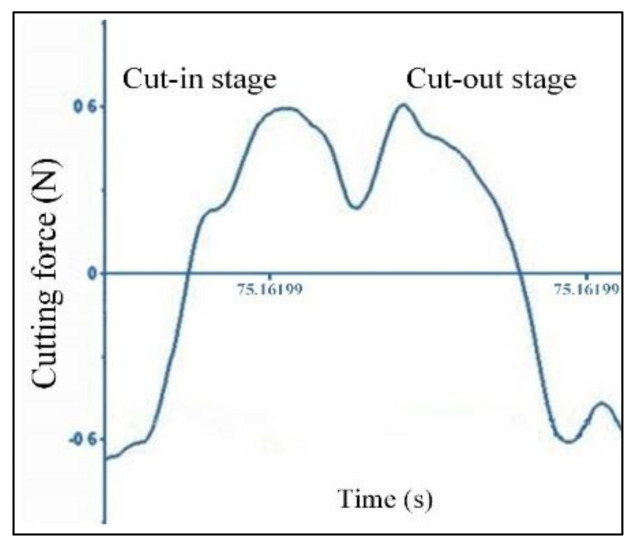
The cutting force signal of single cutting pass (*f_z_* = 0.22 μm, *a_p_* = 5 μm, *n* = 16,000 rpm).

The enlarged diagram of the cutting force signal of single cutting pass at *f_z_* = 0.22 μm is shown in Figure 8. It is seen that the cutting force signal curve of single cutting pass exhibits a crater shape. The theoretical sine profile is not observed in the cutting force signal. With tool rotating, the peak point does not appear at the middle position but appears at the cut-in and cut-out stage. This result also verifies the (*n+2*)th cutting pass, as shown in Figure 5d. The residual material from previous cutting pass leads to the instantaneous undeformed chip thickness in the cut-in and cut-out stage exceeding the minimum undeformed chip thickness, and then the cutting behavior occurs. This corresponds at the peak points in the cut-in and cut-out stage. But, at the middle position, there is no residual material, and the low instantaneous undeformed chip thickness cannot form cutting behavior. It corresponds to the crater on the cutting force signal waveform.

**Figure 9:**
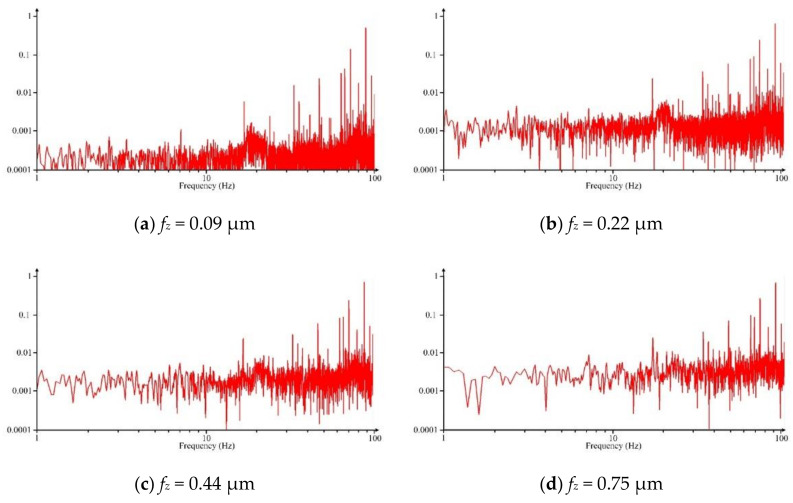

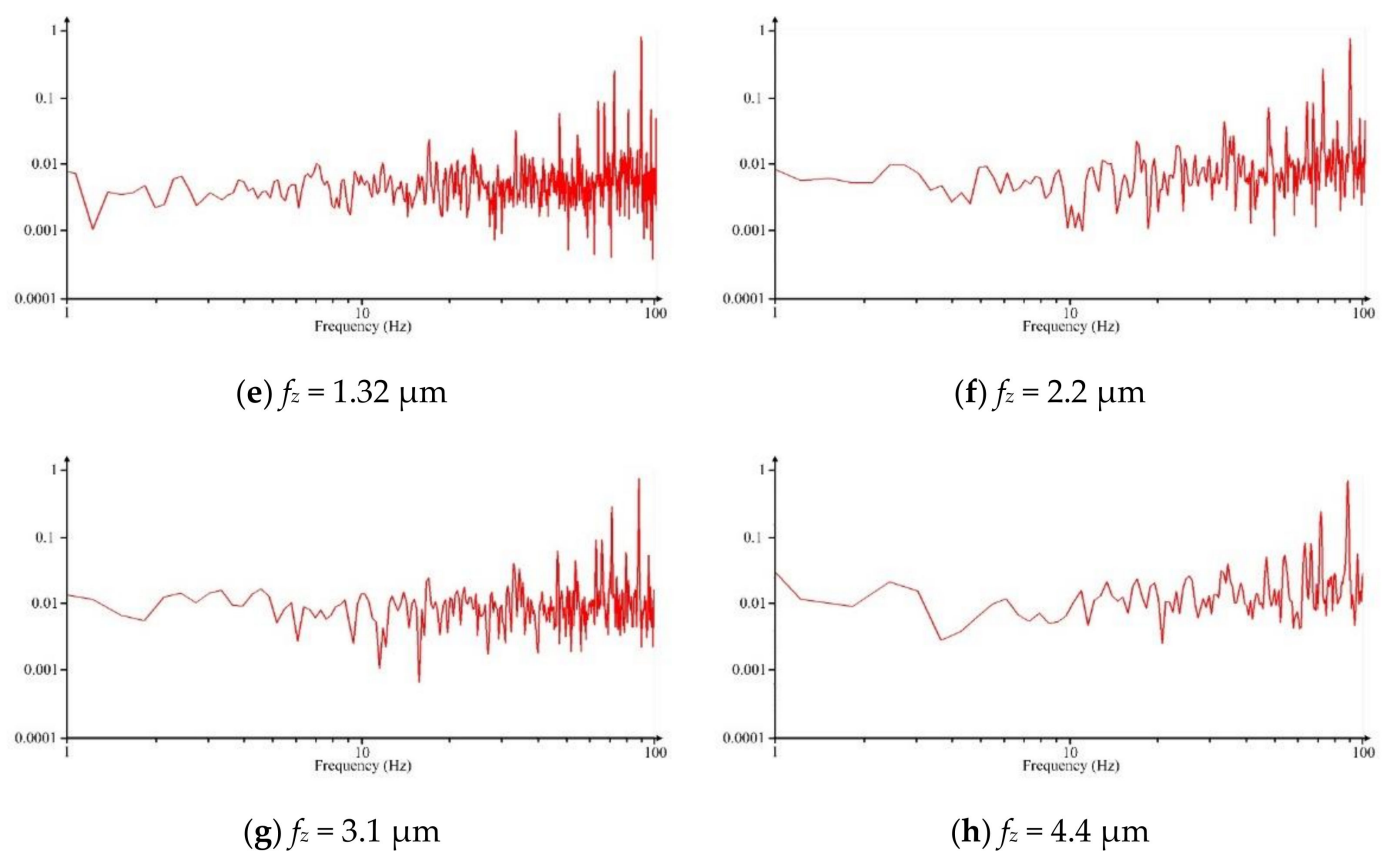
The frequency domain signal at different feed per tooth (*a_p_* = 5 μm, *n* = 16,000 rpm)**.**

Due to the influence of the minimum undeformed chip thickness phenomenon in cutting process, besides the frequencies of spindle rotating, there are other additional frequencies, such as the unstable cutting behavior, the periodic material accumulating, ploughing effect, etc. These additional frequencies, mainly caused by the minimum undeformed chip thickness, usually are smaller than the frequency of spindle rotating. Hence, to observe these additional frequencies, Fourier transform is performed on the recorded cutting force and the frequency domain signal in the range of 100 Hz is shown in Figure 9. It is observed that the characteristic frequency gradually decreases with increasing the feed per tooth. When the feed per tooth is less than the minimum undeformed chip thickness, the characteristic frequency that distributed on frequency domain signal is very extensive and complex, and the cutting process is very unstable, as shown in Figure 9a–c. Once the feed per tooth increases to larger than *h_min_*, the characteristic frequency distribution exhibits a great reduction because the additional frequencies gradually eliminates, as shown in Figure 9e–g. Once the feed per tooth reaches tool cutting edge radius, the characteristic frequency distribution becomes relatively few, as shown in Figure 9h. It indicates that the cutting process becomes very stable.

### 4.3. The Cutting Force, Specific Cutting Energy, and Surface Roughness

**Figure 10:**
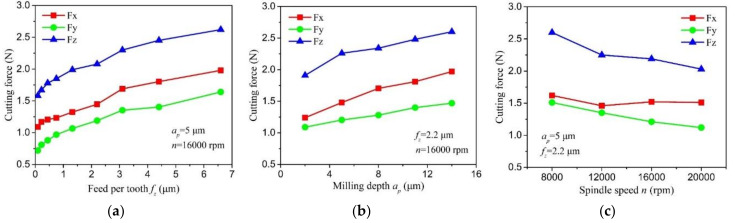
The cutting force curve varies with milling parameters: (**a**) cutting force versus feed per tooth, (**b**) cutting force versus depth of cut, (**c**) cutting force versus spindle speed.

Figure 10 shows the cutting force curve at different milling parameters. Among the components of resultant force, the passive force *F_z_* is the largest, the feed perpendicular force *F_x_* is the middle, and the feed force *F_y_* is the smallest. It is seen that the cutting force exhibits a gradually increase trend with the feed per tooth due to more material is removed during micro milling process. When the feed per tooth increases from 0.09 μm to 6.6 μm, the feed perpendicular force, feed force, and passive force increase from 0.72 N, 1.09 N, and 1.58 N to 1.64 N, 1.99 N, and 2.61 N, respectively. The cutting force shows an approximately linear increase trend with depth of the cut. Once the depth of cut increases from 2 μm to 14 μm, the three components of resultant force increase from 1.09 N, 1.24 N, and 1.91 N to 1.47 N, 1.97 N, and 2.63 N, respectively. With the increase of spindle speed, the cutting force presents a gradually decrease trend, and the decrement of the passive force is relatively larger among the three components of resultant force.

The specific cutting energy is the energy consumption that required to remove a unit volume of material [30]. It is calculated using the following equation, where S is the specific cutting energy, $P_{c}$ is the cutting power, $Z_{w}$ is the material removal rate, $F_{c}$ is the feed perpendicular force, and v is the cutting speed.

(5) $$S=\frac{P_{c}}{Z_{w}}=\frac{F_{c}\times v}{v\times f_{z}\times a_{p}}=\frac{F_{c}}{f_{z}\times a_{p}}$$

**Figure 11:**
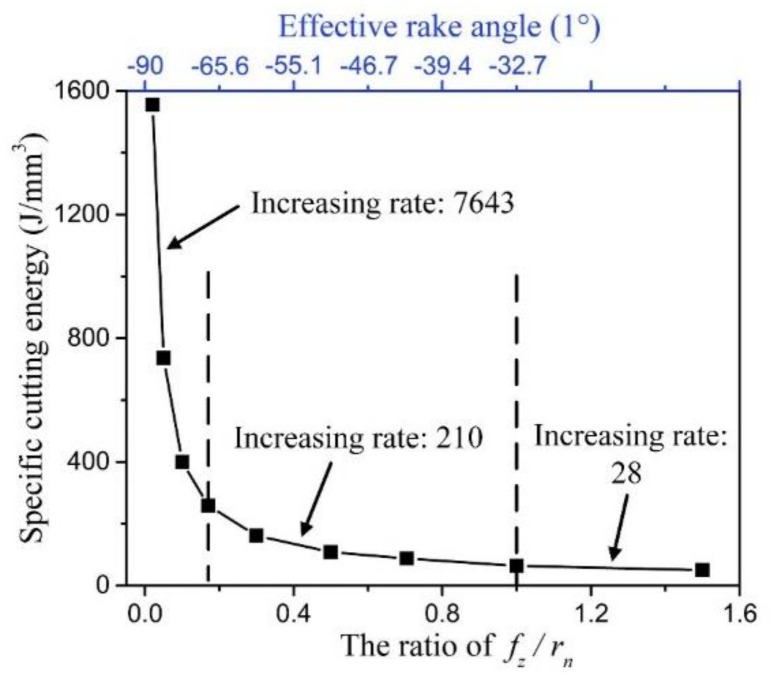
The specific cutting energy curve varies with the ratio of *f_z_*/*r_n_*.

Figure 11 shows the specific cutting energy varying with the ratio of *f_z_*/*r_n_*. The corresponding effective rake angle is marked out on the figure. It is seen that the specific cutting energy curve nonlinearly increases with the decrease of the ratio of *f_z_*/*r_n_* and exhibits obvious size effect. The increasing rate can be reflected in the averaging slope of specific cutting energy curve. Based on the increasing rate, the specific cutting energy curve can be divided into three zones. When the ratio of *f_z_*/*r_n_* is larger than 1, the increasing rate of specific cutting energy curve is 28, and this is the zone of weak size effect. In this zone, the cutting process is stable with the state of positive tool rake angle. And the specific cutting energy is low due to the cutting behavior is dominant during cutting process. Once the ratio of *f_z_*/*r_n_* is less than 1 and larger than 0.17, the increasing rate of specific cutting energy curve is 210, and this is the zone of medium size effect. The cutting process begins to go into the state of negative effective rake angle. The energy consumption gradually increases due to more serious ploughing in cutting process. Once the ratio of *f_z_*/*r_n_* is less than 0.17, the increasing rate rapidly reaches to 7643, and this is named the zone of severe size effect. In this case, the effective rake angle decreases to more negative than the minimum negative rake angle for chip formation, and the whole cutting process goes into the minimum undeformed chip thickness phenomenon. The ploughing factor becomes dominant in cutting process, and it consumes extensive energy. Hence, the specific cutting energy is very large.

**Figure 12:**
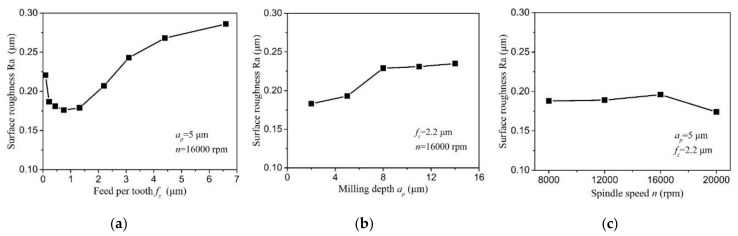
The surface roughness curve varies with milling parameters: (**a**) surface roughness versus feed per tooth, (**b**) surface roughness versus depth of cut, (**c**) surface roughness versus spindle speed.

Figure 12 shows the effect of milling parameters on surface roughness. It is found that the feed per tooth presents the greatest effect on surface roughness Ra. The surface roughness first decreases, and then gradually increases with the decrease of the feed per tooth. The minimum surface roughness is obtained to be 0.176 μm at *f_z_* = *h_min_*. Once the feed per tooth is less than *h_min_*, the cutting state of severe negative rake angle and the dominant ploughing in cutting process greatly deteriorate the machined surface, resulting in the larger surface roughness. When the feed per tooth is larger than *h_min_*, the theoretical residual material, which increases with the feed per tooth, is the key factor that affects surface roughness. Hence, the surface roughness increases with the larger feed per tooth, as well. The surface roughness exhibits a gradual increase trend with the increase of depth of cut. The larger depth of cut can lead to the higher cutting force and then may induce more tool deflection due to the small diameter and weak rigidity of micro end mill. To a certain extent, this factor can slightly increase the surface roughness. With the larger cutting speed, the surface roughness shows a slight decrease. But the effect of spindle speed on surface roughness is relatively little.

## 5. Conclusions

This paper presents an experimental study on the minimum undeformed chip thickness based on effective rake angle in micro milling. The following conclusions can be drawn:

1. The averaging method is proposed to estimate the effective rake angle during micro milling. Once the undeformed chip thickness is less than tool cutting edge radius, the cutting process goes into the cutting state of negative rake angle. The effective rake angle becomes more negative with decreasing the ratio of the undeformed chip thickness to tool cutting edge radius. The minimum undeformed chip thickness of copper is determined as 0.17 *r_n_* based on the effective rake angle curve.
2. The minimum undeformed chip thickness leads to the unstable cutting force signal during micro milling. The drawn envelope of peak forces in cutting force signal becomes uneven with some hump points. The cutting force signal of single cutting pass becomes a crater shape but not the sine profile. And the characteristic frequency distribution on frequency domain signal becomes dense and complex.
3. Based on the increasing rate, the specific cutting energy curve is divided into three zones of the weak, medium, and severe size effect zone. Once the undeformed chip thickness is less than the minimum undeformed chip thickness, the effective rake angle decreases to more negative than the minimum negative rake angle for chip formation. The dominant ploughing effect induces the great energy consumption and deteriorated surface roughness.

## Figures and Tables

**Table 1:** Micro milling parameters.

Parameters	Value
Spindle speed (rpm)	8000, 12,000, 16,000, 20,000
Depth of cut (μm)	2, 5, 8, 11, 14
Feed per tooth (μm)	0.09, 0.22, 0.44, 0.75, 1.32, 2.2, 3.1, 4.4, 6.6
